# Water-Deprived Parasitic Wasps (*Pachycrepoideus vindemmiae*) Kill More Pupae of a Pest (*Drosophila suzukii*) as a Water-Intake Strategy

**DOI:** 10.1038/s41598-019-40256-8

**Published:** 2019-03-05

**Authors:** Cherre Sade Bezerra Da Silva, Briana Elizabeth Price, Vaughn M. Walton

**Affiliations:** 1 Department of Horticulture, Oregon State University, 4017 Agricultural and Life Sciences Building, Corvallis, OR 97331 USA; 2 Present Address: Embrapa Algodão, Rua Oswaldo Cruz 1143, Campina Grande, PB, 58428-095 Brazil

## Abstract

Most organisms must ingest water to compensate for dehydration. In parasitic wasps, the importance of water and the behaviors driving its consumption are poorly understood. Here, we describe a water-intake strategy of *Pachycrepoideus vindemmiae*, a parasitoid of spotted-wing drosophila (SWD, *Drosophila suzukii*). Longevity measurements indicated that *P. vindemmiae* benefits from drinking water and from host-feeding on the water-rich hemolymph of SWD pupae. After exposing wasps to different water regimens, we observed increased host-feeding in water-deprived wasps despite honey availability. This resulted in greater SWD mortality because the host-feeding process killed the pupae, and because wasps that engaged in greater host-feeding parasitized more hosts. Behavioral observations showed that the host-feeding time of water-deprived wasps doubled compared to water-fed individuals. Host-feeding did not affect parasitoid offspring mortality. We conclude that *P. vindemmiae* benefits from ingesting water and that it host-feeds on SWD pupae as a water-intake strategy. These are interesting findings not only because water has rarely been reported as a critical nutrient for adult parasitoids, but especially because preying for the purpose of hydration is not a common strategy in nature. This strategy enhances parasitoid survival and reproduction, with positive consequences for its host-killing capacity and potential as a biocontrol agent.

## Introduction

Water is an essential nutrient for all forms of life, including insects^1,2^. Through respiration and other metabolic processes such as excretion, insects lose water to the environment. Hence, survival and normal behavior demand constant water replenishment^3–7^.

Within Hymenoptera, bees and non-parasitic wasps are known to forage for free (liquid) water, and its importance for providing their larvae, nest construction, and thermoregulation is well known^8–10^. Conversely, free water consumption by parasitic wasps has been reported few times^4,11^. Its effects on parasitoid biology have rarely been explored and it is unclear whether free water would affect the wasps’ physiology^12^. Indirect water consumption via sugary solutions (e.g., nectar and honeydew) and host-feeding (*i.e*., feeding on hemolymph or tissues of the host) is considered very common^11–16^. These meals increase the longevity, fecundity, sex ratio, and flight capacity of parasitic wasps^13,15,17^. However, while most of those benefits have been attributed to the energetic fraction of the meals, which is constituted of sugars, glycogen, amino acids, proteins, and lipids^15,18–20^, the contribution of water within these meals has been considered low or inexistent^12,21–26^, which is perhaps an oversight, since water makes up to 88–98% of floral nectar^27,28^, 89–94% of insect honeydew^29,30^, and 90–95% of insect hemolymph^18,31^.

The lack of studies demonstrating the benefits of water intake to parasitic wasps has likely done little to inspire research on strategies employed by these organisms in order to ingest water. In the present study, lifespan measurements demonstrated that females of *Pachycrepoideus vindemmiae* (Hymenoptera: Pteromalidae) – a pupal ectoparasitic wasp of the invasive spotted-wing drosophila (SWD, *Drosophila suzukii*) and many other dipteran families^32,33^ – benefit from ingesting free water as well as the water-rich hemolymph of SWD pupae (*i.e*., host-feeding). We then questioned whether *P. vindemmiae* would perform host-feeding as a strategy for water intake. Physiological and behavioral studies confirmed this hypothesis and showed that in addition to extending lifespan, this strategy increases the reproduction and total host-killing capacity of *P. vindemmiae* without compromising offspring survival.

## Results

**Figure 1:**
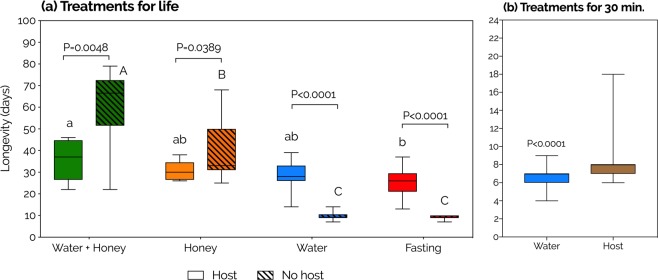
Effects of water, honey, and hosts on the longevity of females of *Pachycrepoideus vindemmiae* (Hymenoptera: Pteromalidae) reared on pupae of spotted-wing drosophila (*Drosophila suzukii*). (**a**) Uninterrupted provision of treatments. N = 13 (water), 12 (honey), 14 (water + honey), 10 (no water, no honey), 11 (water + hosts), 14 (honey + hosts), 10 (water + honey + hosts), and 13 (no water, no honey + hosts) mated individual wasps. (**b**) 30-minute provision of treatments. N = 94 (water) and 70 (hosts). Distinct lowercase and uppercase letters indicate significant differences among water/honey regimens within host-provided and host-deprived females, respectively (Kruskal-Wallis followed by Dunn’s method). P-values according to either unpaired two-tailed t-test or Mann-Whitney U test.

To determine whether *P. vindemmiae* ingest free water and its potential impact on their life-history traits, we exposed adult female wasps to (1) water, (2) honey, (3) water + honey (independent sources), or (4) fasting (no water, no honey), in the presence and absence of fresh hosts (SWD pupae), for their entire lives (N = 11, 14, 10, and 13 in host presence; and 13, 12, 14, and 10 in host absence, respectively for water, honey, water + honey, and fasting). Honey was included in the experimental design as a low-water energy source^34^ to control for energy-deprivation. In host absence, water + honey extended female longevity more than either nutrient separately, significantly surpassing water by more than 6-fold and honey by more than 2-fold (Fig. 1a), giving the first demonstration that host-deprived females of *P. vindemmiae* seek and ingest free water. In host presence, no significant difference was observed among the water, honey, and water + honey treatments, but the latter was the only regimen that significantly increased longevity relative to fasting (Fig. 1a), indicating that host-provided females of *P. vindemmiae* also seek and drink free water (as well as sugars). In honey-fed wasps (honey, and water + honey), the presence of hosts dramatically shortened female longevity relative to host absence, exposing a trade-off between longevity and reproduction in sugar-rich environments. The opposite effect was observed in the honey-deprived individuals (water alone, and fasting), demonstrating that females of *P. vindemmiae* host-feed on pupae of SWD (Fig. 1a). A single host-feeding bout significantly increased female longevity by 1.3 day relatively to water-fed wasps (host N = 70, water N = 94, P < 0.0001, Fig. 1b).

**Figure 2:**
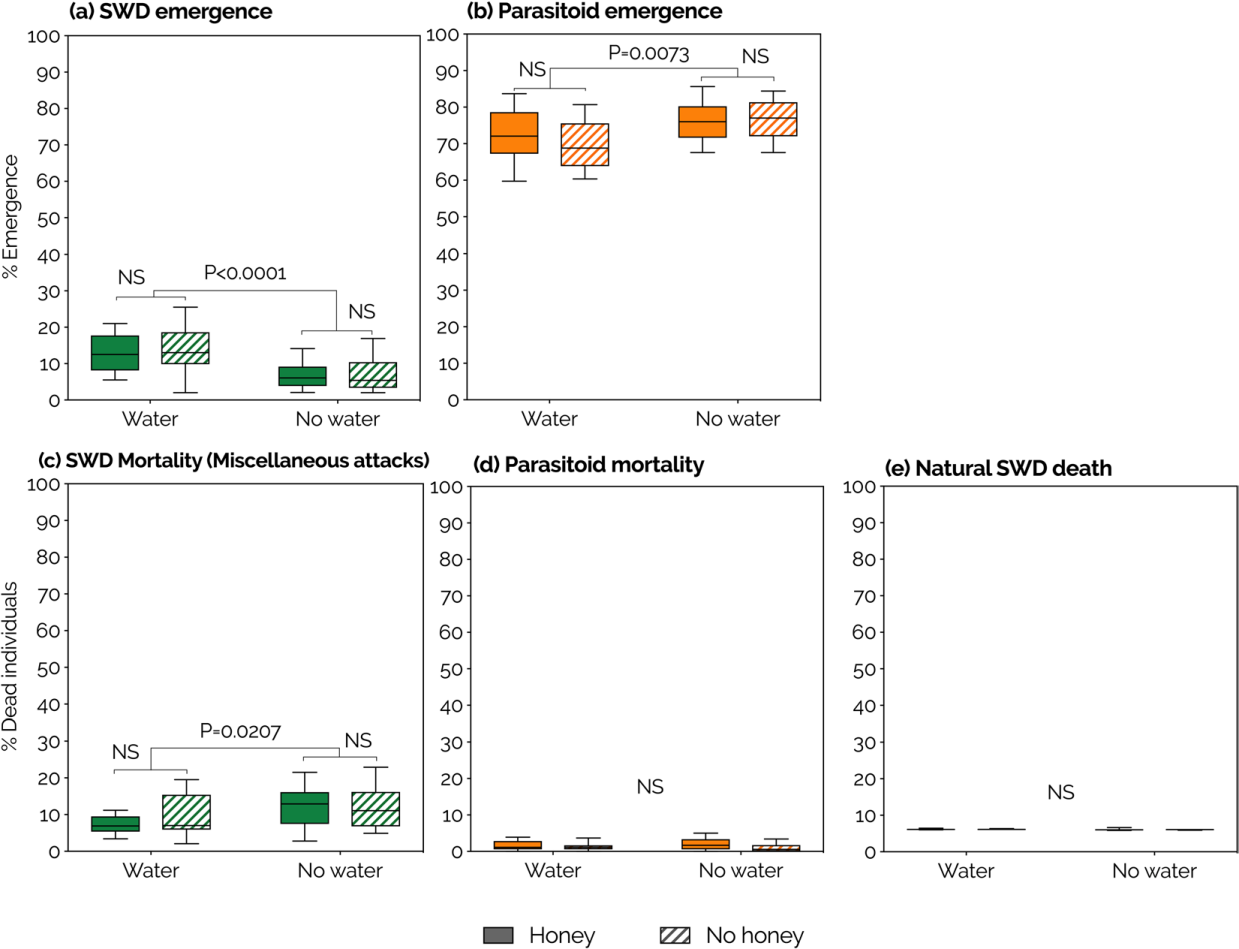
Effects of water and honey on life-history traits of *Pachycrepoideus vindemmiae* (Hymenoptera: Pteromalidae) reared on pupae of spotted-wing drosophila (SWD, *Drosophila suzukii*). (**a**) SWD emergence – percent flies that emerged as adults. (**b**) Parasitoid emergence – percent SWD pupal cases that gave rise to adult parasitoids. (**c**) SWD mortality caused by host-feeding + nonreproductive effects^35^ of *P. vindemmiae* – percent dead SWD pupae (excluding parasitism) minus natural SWD death. (**d**) Parasitoid mortality – percent SWD pupal cases containing a dead parasitoid. (**e**) Natural SWD death – percent SWD pupal cases not exposed to parasitism and that did not give rise to adult flies. P-values were calculated by Two-Way ANOVA. N = 11 (water), 14 (honey), 10 (water + honey), and 13 (no water, no honey) mated individual wasps; NS = no significant effect.

To elucidate whether water-deprivation increases host-feeding in *P. vindemmiae* females, we measured the emergence rate of adult SWD from pupae exposed to wasps under the four water/honey regimens previously described. We observed a strong reduction of SWD emergence in water-deprived wasps relative to their water-fed counterparts, irrespective of honey availability (P < 0.0001, Fig. 2a). Further analysis showed that such reduction was caused by clear increments in both offspring production (P = 0.0073, Fig. 2b) and miscellaneous attacks (P = 0.0207, Fig. 2c) of water-deprived wasps. Miscellaneous attacks include host-feeding and nonreproductive effects^35^. Increased host-feeding was then indirectly identified because it contributes to offspring production while nonreproductive effects do not^35^ (see Discussion and Methods for details). No significant effect of water or honey was found on parasitoid offspring mortality (Fig. 2d) nor on natural SWD death (Fig. 2e).

**Figure 3:**
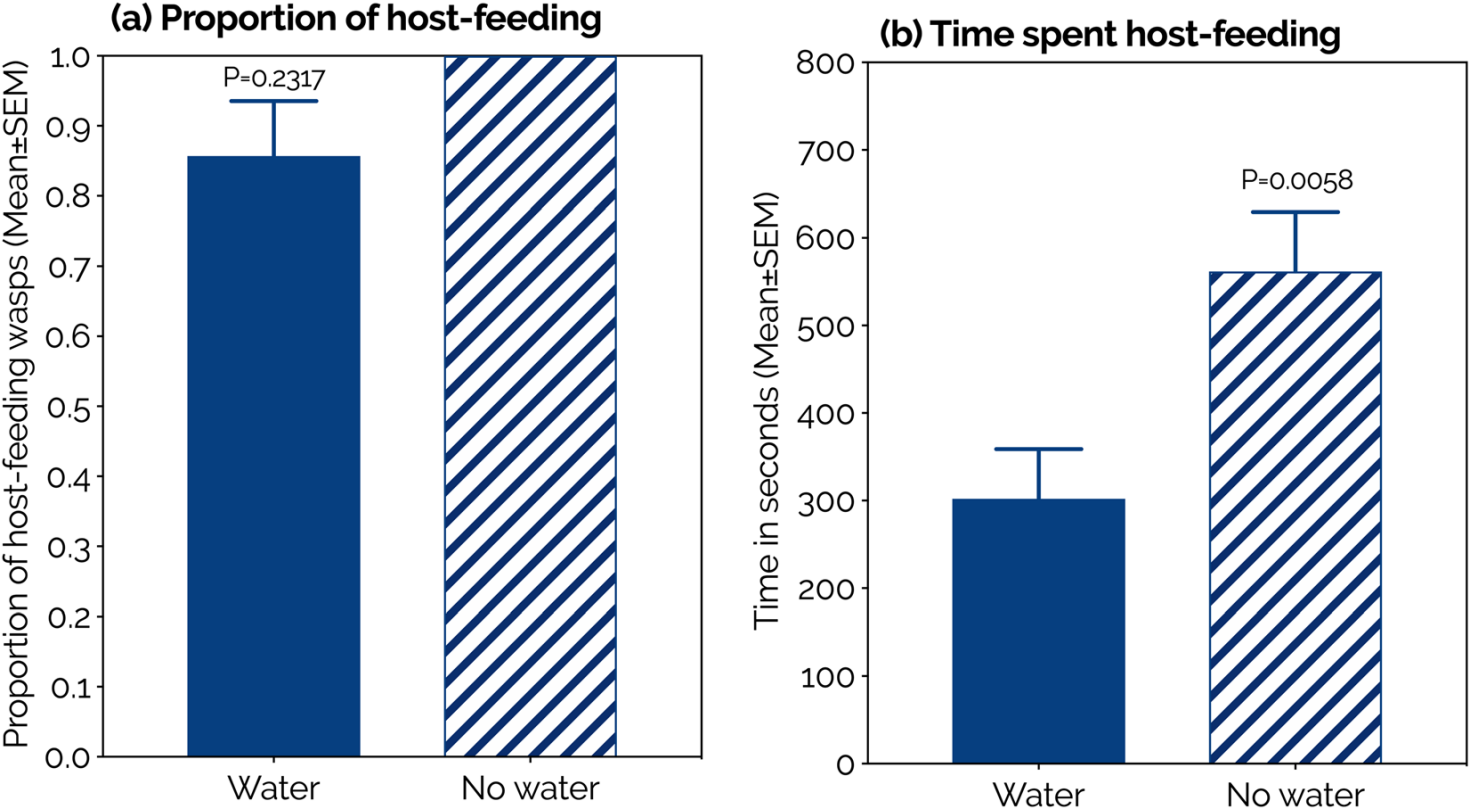
Effects of water on the host-feeding behavior of *Pachycrepoideus vindemmiae* (Hymenoptera: Pteromalidae) reared on pupae of spotted-wing drosophila (SWD, *Drosophila suzukii*). (**a**) Proportion of female wasps displaying host-feeding behavior, Mann-Whitney U test. (**b**) Length of host-feeding (seconds), unpaired two-tailed t-test. N = 20 (water) and 21 (no water) mated individual wasps.

In order to better understand how water-deprivation affected the host-feeding behavior of *P. vindemmiae*, we filmed single water-deprived and water-fed wasps in arenas containing SWD pupae. We found no significant difference between the two groups regarding frequency of host-feeding, and 85–100% of the females practiced this behavior irrespective of the water regimen (Fig. 3a, Supplementary Video S1). Nonetheless, water-deprived wasps spent significantly more time host-feeding than their water-fed counterparts (water N = 20, no water N = 21, P = 0.0058, Fig. 3b).

## Discussion

In our initial experiment, the consumption of water + honey by host-deprived females of *P. vindemmiae* increased their lifespan to a much greater extent than the sum of both nutrients offered separately, revealing a powerful synergistic effect of water + honey as a suitable nutrient regimen. Moreover, this was the only diet that extended female longevity in comparison to fasting when hosts were present. These results clearly show that females of *P. vindemmiae* seek and consume free water, benefiting from it as long as a sugar source (honey) is available. While our findings strongly contrast with the literature^12,21–26^, such divergence is likely a result of two factors. First, these studies were often designed to assess the response of parasitoids to sugary solutions rather than water. The latter requires an experimental design that tests pure water and a negative control (total fasting) in the same study, as we did in ours. Second, relative humidity (R.H.) was often ignored in these studies. When water or an aqueous solution is offered to parasitoids, water vapor dissipates into the immediate environment and increases the R.H. relative to a control that received no water or aqueous solution, and such R.H. divergences between treatment and control affect the parasitoid’s physiology^36,37^. The fact that *P. vindemmiae* females were able to feed directly on pure honey, which contains only 16–20% moisture^34^, indicates that this species can feed on other viscous sugar sources such as concentrated honeydew, plant secretions, and fruit juices in the field. Moreover, *P. vindemmiae*’s ability to drink free water, as shown in our study, may improve the uptake of highly viscous sugar sources such as honey^12^. This may explain why water + honey caused the longest longevities and suggests that by drinking free water the wasps can better exploit viscous sugar sources in the field.

Although non-host diets (water/honey) affected female lifespan independently of host availability, pairwise comparisons revealed a much weaker impact on host-provided individuals. Hence, to some extent, the presence of hosts stabilized female longevity relative to host absence. Interestingly, this effect was triggered by two distinct mechanisms in a honey-dependent manner. First, individuals that consumed honey lived longer in the absence of hosts compared to when hosts were present, revealing a trade-off between longevity and reproduction in sugar-rich environments. In this scenario, while host-deprived wasps allocated all the energy obtained from honey meals exclusively to somatic maintenance, their host-provided counterparts invested it in somatic maintenance as well as in egg maturation, host foraging, and oviposition, processes with a high nutrient demand, thus explaining the reduced lifespan of females that had a chance to reproduce^15,38,39^. Second, honey-starved individuals lived at least 2.5-fold longer in the presence than in the absence of hosts, providing indirect evidence that females of *P. vindemmiae* host-feed on pupae of SWD and benefit greatly from it in sugar-poor environments. A bout of feeding on a single host was enough to extend female survival by more than one day. In addition, host-feeding improved female fecundity (as will be discussed below), indicating that wasps allocated nutrients obtained exclusively through host-feeding to both somatic maintenance and reproduction. This ability confers resource flexibility and mitigates the parasitoids’ dependency on non-host food sources including nectar, honeydew, fruit juices, and other sugary substances, whose lack is seen as one of the causes of failure of arthropods in biological control programs^40–43^.

Given that *P. vindemmiae* host-fed on SWD pupae, one may hypothesize that the host-provided wasps neither seek nor consume water and/or honey, thus explaining the much weaker impact of non-host diets on longevity in host presence compared to host absence. However, the higher longevity observed in water + honey relative to fasting demonstrates that even when plenty of hosts are available, the wasps still seek, feed, and benefit from independent water/sugar sources. This is an indication that provision of non-host water and energy sources, although not critical for survival or reproduction of host-feeding individuals of *P. vindemmiae*, can contribute to its biocontrol performance in the field. Exploiting those sources could be a strategy to avoid host depletion since these may lose quality after being fed on and will either produce smaller (thus less fit) offspring or will not be used for oviposition at all^15^. The wasps that displayed enhanced host-feeding also showed the highest offspring emergence, which is a function of fecundity and offspring mortality. The latter was unaffected by the four water/honey regimens and thus cannot account for increased offspring emergence. This leaves fecundity as the cause of such an increment and hence establishes a connection among water availability, host-feeding, and fecundity. These findings seem very plausible if we consider that *P. vindemmiae* is synovigenic and lay anhydropic eggs^44,45^, hence losing much water through oogenesis and oviposition. Because hemolymph is rich in water, amino acids, proteins, carbohydrates, and lipids^15,18–20^ – basically the same components that form the contents of insect eggs^44,46^ – it is not surprising that increased host-feeding resulted in higher fecundity.

Miscellaneous attacks of *P. vindemmiae* on SWD pupae were increased in water-deprived relative to water-provided females, independent of honey. As explained, miscellaneous attacks include both host-feeding and nonreproductive effects^35^. We inferred the role of host-feeding on that increment by considering the following:

- Water deprivation supports higher fecundity than water provision (Fig. 2b,d);
- Each host-fed pupa adds 2 mature eggs to *P. vindemmiae*’s fecundity^47^;
- Host-feeding contributes to fecundity while nonreproductive effects do not^35^.

So the increased fecundity observed in water-deprived females demanded increased number of host-fed SWD pupae. Hence, even though host-feeding was not observed directly, we do have evidence that host-feeding was increased and is the proximate cause of the increased miscellaneous attacks in water-deprived wasps, which culminated with SWD mortality. This was demonstrated when each wasp had the opportunity to exploit and host-feed on a total of 150 hosts over 5 consecutive days. Our evidence is nevertheless limited to showing that host-feeding is increased in water deprivation relative to water provision, and do not imply that host-feeding is the only type of miscellaneous attack practiced by the parasitoids. By enhancing both host-feeding and fecundity, water-deprivation increased the overall host-killing capacity of *P. vindemmiae*, resulting in half the SWD emergence of that observed in water-fed individuals. This has important implications to mass rearing and the use of this parasitoid in biological control programs as it demonstrates that by manipulating water availability it is possible to improve parasitoid yield and total pest-killing capacity.

The higher host-feeding rates in water-deprived wasps shows that such trophic behavior was employed as a strategy of water intake. Host-feeding rates were enhanced even in the presence of a highly energetic and phagostimulant nutrient such as honey^48^, reinforcing that the water-deprived wasps host-fed primarily for hydration rather than energetic purposes. This strategy was confirmed in our behavioral assay, where each wasp had the opportunity to host-feed for 30 minutes. Here, increased host-feeding duration was observed directly in water-deprived relative to water-provisioned wasps, i.e., thirsty females consumed more SWD hemolymph. The thirsty wasps could have ignored hemolymph as a water source, but they did not. They made the decision to keep drinking hemolymph for a period 2x as long as their water-fed counterparts. That decision was strategic and not random as it was observed more in water-deprived than in water-provisioned females, implying that wasps spend more time host-feeding as a water-intake strategy as well. Therefore, our study shows that both the rate and duration of host-feeding are increased as water-intake strategies by thirsty females of *P. vindemmiae*. It is true that in our behavioral experiment only the duration (Fig. 3b) and not the rate (Fig. 3a) of host-feeding was incremented under water deprivation. However, we tested 6–7-day-old wasps that were completely naïve to sugar and hosts. Hence, those 30 minutes of contact with SWD pupae were their first opportunity ever to access energy, which is widely known as one of the purposes of host-feeding^13,49,50^. So it was expected that most wasps practiced host-feeding under such conditions of hunger, independent of the water regimen. These are interesting findings not only because water has rarely been reported as a critical nutrient for adult parasitoids, but especially because predation for the purpose of hydration is not a common strategy in nature. Indeed, we are unaware of other reports on predators or parasitoids attacking, killing, and consuming prey for the purpose of quenching their thirst. Importantly, by concluding that thirsty females of *P. vindemmiae* host-feed on hemolymph of SWD pupae as a strategy to ingest water we do not negate the contribution of the other constituents of the hemolymph such as sugars, amino acids, and lipids, as indicated in our behavioral experiment and the above-mentioned literature.

In field, free water is often a ubiquitous resource (morning dew, rain drops, pooled water, irrigation, etc.). Our study showed that *P. vindemmiae* females not only seek free water, but they also benefit from consuming that abundant resource even when hosts are available. On the other hand, our findings also demonstrated that *P. vindemmiae* females displayed enhanced host-feeding when hosts were abundant relative to free water, and that this behavior was accompanied by survival and reproductive benefits. This implies that host-feeding as a water-intake strategy is adaptive under high abundance of hosts and low availability of free water. Additionally, it shows that the host-feeding potential of *P. vindemmiae* as a SWD mortality factor will likely be highest in arid/semiarid environments or during periods of drought.

Here, we show that females of *P. vindemmiae* seek out and consume free water, and that free water supports extended longevity. We also show that thirsty parasitoids host-feed as a water-intake strategy, and that this strategy is adaptive in dry environments as it supports extended longevity and higher fecundity during a life stage when much of the physiological water demand is devoted to reproduction. This flexibility of hydrating directly (by drinking free water) and indirectly (through host-feeding) increases the accessibility of this important nutrient to *P. vindemmiae*. Additionally, when a female parasitoid accesses water, food, and an oviposition site from a single source (SWD pupae) she saves time and energy, both of which can be allocated to reproduction (i.e., increase fitness). Finally, host-feeding as a water-intake strategy enhances *P. vindemmiae*’s total host-killing capacity in water-deprived environments. The rarely discussed strategy of drinking the host’s hemolymph to hydrate is expected to be widespread in parasitic wasps as it is greatly adaptive in species whose adult females need water and food for egg maturation, practice host-feeding, and are exposed to high abundance of hosts relative to free water. Both synovigeny and host-feeding are extremely common in parasitic wasps^51,52^, and meteorological drought is a relatively common phenomenon across the globe^53^. Future studies should investigate whether pro-ovigenic species also employ this strategy, and how environmental factors such as R.H. affect it.

## Methods

### Insects

Colonies of both *D. suzukii* and *P. vindemmiae* were held in a climate-controlled chamber (24.1 ± 0.4 °C, 62 ± 8% R.H., and 14:10 L:D photoperiod). SWD colonies have been maintained by the laboratory since 2009 from insects provided by the USDA-ARS (Corvallis, OR, USA); wild-caught insects from the Willamette Valley and Columbia River Gorge of Oregon have been introduced periodically to maintain genetic diversity. SWD larvae were reared on a cornmeal diet and adults were fed a 10% sucrose solution and brewer’s yeast^54^. Colonies of *P. vindemmiae* have been maintained in the laboratory since 2013 from adults emerged from SWD pupae in sentinel traps placed in agricultural and wild vegetation areas of the Willamette Valley and Columbia River Gorge of Oregon^55^. Adult *P. vindemiae* were kept in a Bugdorm mesh cage (32.5 × 32.5 × 32.5 cm) (Bioquip Products, Rancho Dominguez, CA, USA). A Petri dish (9 × 1.5 cm) (Corning Falcon™) containing streaks of pure honey, and a pint container with a sponge soaked in de-ionized water were available within the cage to provide food and moisture. Another pint container filled with 1,000–2,000 pupae of *D. suzukii* (1–2 days old) was inserted into the parasitoid cage weekly and exposed to parasitism by *P. vindemiae* for 24 h. The container was then withdrawn from the cage; any remaining wasps within it were aspirated and released back into the parasitoid cage. The container with pupae was covered with a screen lid and held in the climate-controlled chamber until parasitoid emergence. Emerged adults were transferred to a Bugdorm cage to renew the cycle.

### Effects of water and honey on life-history traits

Newly emerged (<24 h) adults of *P. vindemmiae*, water-, honey-, and host-deprived, were allowed to mate in a Bugdorm cage for 2 d. On day 3, the females were individualized in flat tubes (2 × 9 cm) (Genesee Scientific, San Diego, CA, USA) and offered water alone, honey alone, water + honey (independent sources), or fasting (no water, no honey), in the presence or absence of hosts (SWD pupae) (N = 11, 14, 10, and 13 in host presence; and 13, 12, 14, and 10 in host absence respectively for water, honey, water + honey, and fasting) (Supplementary Fig. S1a). Honey was offered as a small drop placed on the tube wall. Water was provided in a thin (3 mm wide) piece of filter paper protruding through a hole made at the tip of a 2-mL Eppendorf tube filled with de-ionized water. The total length of the paper was 40 mm, but only *ca*. 3 mm protruded from tube. The base of the Eppendorf tube was attached to the bottom of the flat tube with modeling compound (Play-Doh^®^, Hasbro, Pawtucket, RI, USA) to prevent it from shifting (Fig. S1a,b). To control for potential R.H. differences between water-provided and water-deprived tubes, the same water source was provided within all tubes independent of the water/honey regimen. However, for the water-deprived treatments, the top of the Eppendorf tube (including the protruding paper) was covered with a screened cap to prevent wasps from accessing the moistened paper, while allowing moisture to dissipate within the flat tube (Fig. S1c,d). For the host-provided group, 30 young (<2 days old) SWD pupae were offered per female wasp per day until death (Fig. S1b). This number is 2–6x higher than the daily parasitism capacity of *P. vindemmiae* previously reported in the literature^47,56^. Before exposure to females, the pupae were gently rinsed in de-ionized water to remove cornmeal medium and larval excrement, and carefully transferred to a piece of paper towel (2 × 2 cm) (Capri, Cascades Tissue Group – Sales Inc., Eau Claire, WI, USA) with a fine camel brush and de-ionized water. Pupae-carrying paper was allowed to dry at room temperature for 1 h. Each day, 5–8 groups of 30 SWD pupae that had not been exposed to parasitism served as a control for natural SWD death (ND).

Wasp deaths were recorded daily; supplies of water and honey were renewed as needed. The 30 wasp-exposed pupae from each tube were transferred to a new flat tube caped with a cotton plug and stored in the walk-in chamber (24.1 ± 0.4 °C, 62 ± 8% R.H., and 14:10 L:D photoperiod) until adult emergence. Emerged flies and parasitoids were starved to death and counted under a stereomicroscope (Leica S8AP0, Leica Microsystems Inc., Buffalo Grove, IL, USA). SWD pupal cases that did not contain emergence holes were dissected for evidence of parasitism (dead *P. vindemmiae* immature). Host-feeding was assessed indirectly based on the rate of miscellaneous attacks of *P. vindemmiae* on SWD pupae. Miscellaneous attacks are constituted by host feeding and nonreproductive effects (aborted parasitism, pseudoparasitism, and mutilation)^35^. They were calculated as MA = (P-ND)/30 * 100, where MA = miscellaneous attacks, P = pupal cases containing neither an emergence hole nor a dead parasitoid, and ND = natural SWD death, as described earlier. Host-feeding was then inferred by comparing SWD death from offspring production (parasitism) with death by miscellaneous attacks, e.g., an elevated offspring production in water-deprived wasps implies increased host-feeding in the same wasps since host-feeding contributes to offspring production while nonreproductive effects do not^35^. Parasitoid mortality was based on SWD pupal cases containing dead immature parasitoids. To eliminate the effect of adult aging as well as the variation in the total of hosts that were offered to each female (total hosts = 30 * longevity in days), all calculations were based exclusively on wasps aged 4–8 days old. Three-day-old individuals were excluded because newly emerged females already carry an egg load^47^. Older females were excluded in order to homogenize their chronological conditions, and also because their differences in longevity caused variations in the total number of hosts offered to each female, which couldn’t be corrected for by simply applying proportions given the loss of host-killing potential as females age^49,57^.

### Effect of a single host-feeding bout on longevity

To determine how long a single host-feeding bout extends *P. vindemmiae* longevity, we let young water-deprived female wasps feed *ad libitum* on one SWD pupa each and daily monitored the wasps’ survival. *P. vindemmiae* females (2 d old, mated, water-, honey-, and host-deprived) were isolated in #00 gelatin capsules, provided with a single 2-d-old SWD pupa host for 30 minutes, and then starved to death. In a preliminary assay, 30 minutes was enough time for a female to drill a hole in the host’s pupal case, feed from it, and then leave the host. The 30-minute countdown started when each female approached the host. After this period, each female was individually transferred to a flat tube and deprived of water, honey, and hosts. As a control group, water was offered in flat tubes (as described in the previous assay) for 30 min., then the wasps were isolated in new flat tubes and starved to death. All adults were kept in the climate controlled walk-in chamber under the conditions previously described. Deaths were recorded daily.

### Effect of water on host-feeding behavior

Female wasps (2-d old) were deprived of honey and hosts in a flat tube containing a water source. A control group was kept under the same conditions, except that the water source was covered with a screen cap to prevent the wasps from consuming water, as described in the first assay. After 4 to 5 days, females were released individually into an arena containing 3 SWD pupa; their host-feeding behavior was filmed for 30 minutes under a stereomicroscope coupled to a digital camera (Canon EOS Rebel T5i). The arena was formed by a piece of Play-Doh^®^ where the 3 host pupae where equidistantly planted with anterior spiracles pointing up. The PlayDoh^®^ piece was attached to a straightened wire paper clip transversally inserted into a 50 mL deli cup. One of the wire tips remained long and bended enough to be manually rotated; as it rotated so did the attached PlayDoh^®^, giving us ample control over the insects’ position to optimize the angle of the digital recording (Supplementary Fig. S2). The number of females that fed on these pupae was recorded, as was the duration of such behavior. Host-feeding was considered to have begun from the moment when the female’s mouthparts contacted the host’s hemolymph until the point at which she removed her mouth parts from the hemolymph.

### Statistical analysis

After confirmation of normality (Shapiro-Wilk, D’Agostino & Pearson, and Kolmogorov-Sminorv’s tests) (Supplementary Table S1), and homoscedasticity (Barllet’s and Brown-Forsythe’s tests) (Supplementary Table S2), Two-Way ANOVA followed by Tukey’s test (α = 0.05) was applied to test the effects of water (presence and absence) and honey (presence and absence) on SWD emergence, parasitoid emergence, SWD mortality by miscellaneous attacks, parasitoid offspring mortality, and natural SWD death. Either we failed in attempts to normalize longevity data or the treatments’ variances were significantly different from each other. Hence, Kruskal-Wallis’s test followed by Dunn’s method was applied to determine the effect of water/honey regimen (water + honey, water, honey, and fasting) on the longevity of host-provided and host-deprived females in the first assay. Unpaired *t*-test (when variances were not significantly different) or Mann-Whitney U test (when variances were significantly different) were applied to compare two treatments in the first, second and third assays. All analyses were performed using GraphPad Prism version 7.0b for Mac OS X (GraphPad Software, La Jolla, CA, USA).

## Supplementary information

Supplementary Figures S1 and S2, Supplementary Tables S1 and S2, Legend of Supplementary Video S1

Supplementary video S1

## Data Availability

The datasets generated during and/or analyzed during the current study are available from the corresponding author upon reasonable request.
