# Supplementary material for: Water-Deprived Parasitic Wasps (Pachycrepoideus vindemmiae) Kill More Pupae of a Pest (Drosophila suzukii) as a Water-Intake Strategy
Source: Sci Rep. 2019 Mar 5;9:3592. doi: 10.1038/s41598-019-40256-8 (PMC6400901; doi:10.1038/s41598-019-40256-8)
Supplement: Supplementary file 1 — Supplementary Figures S1 and S2, Supplementary Tables S1 and S2, Legend of Supplementary Video S1 [file 41598_2019_40256_MOESM1_ESM.pdf]

Water-Deprived Parasitic Wasps (*Pachycrepoideus vindemmiae*) Kill More Pupae of a Pest (*Drosophila suzukii*) as a Water-Intake Strategy

Cherre Sade Bezerra Da Silva*[1], Briana Elizabeth Price[1,2], Vaughn M. Walton[1,3]

*Corresponding author. Current affiliation: Embrapa Algodão. Address: Rua Oswaldo Cruz 1143, Campina Grande, PB, Brazil, 58428-095. E-mail: entomologista@gmail.com, Orcid: 0000-0001-9415-6337

[1]Department of Horticulture, Oregon State University, 4017 Agricultural and Life Sciences Building, Corvallis, OR 97331, USA. Phone: +1 (541) 737 3913

[2]E-mail: priceb@oregonstate.edu

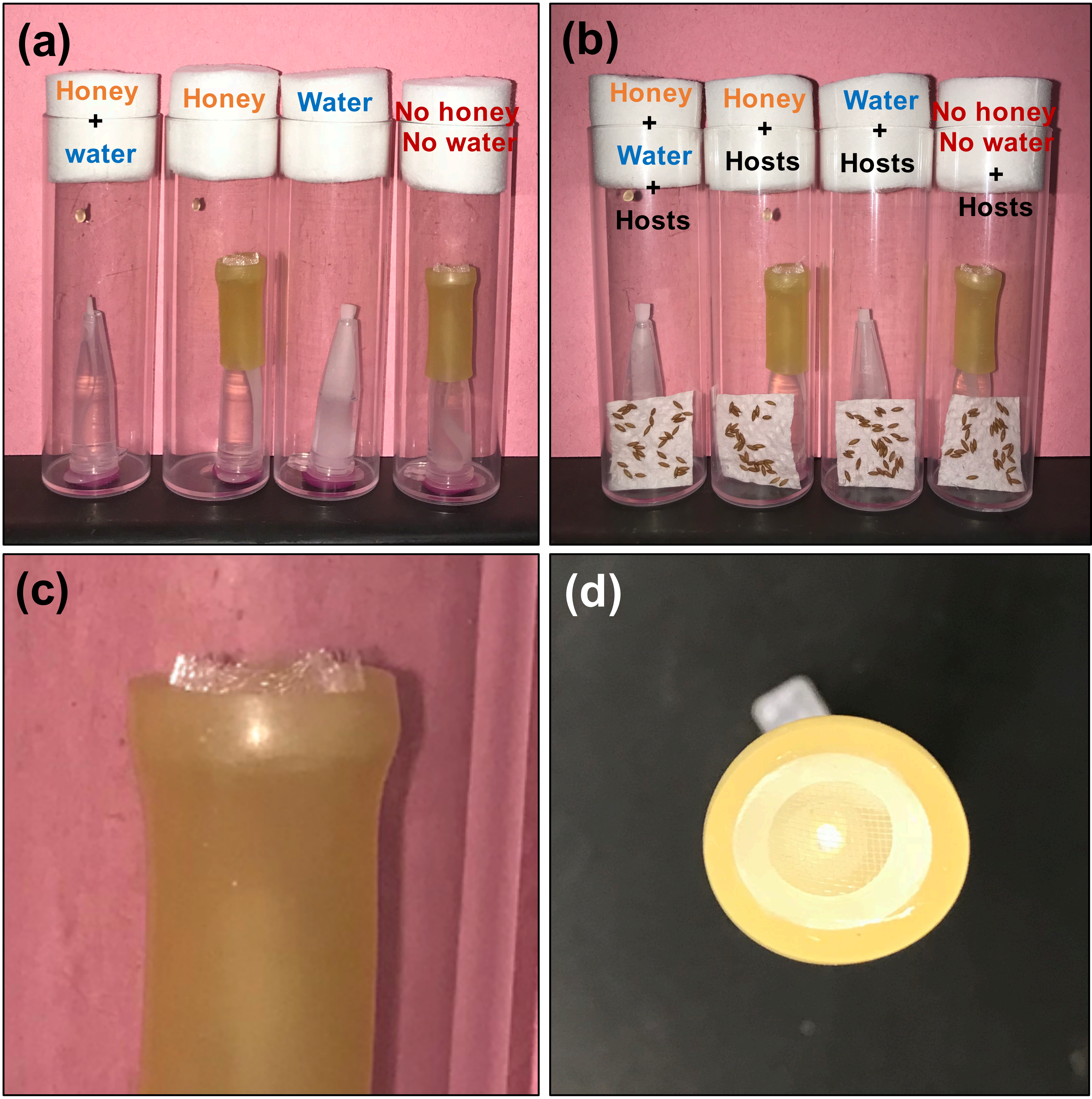

Supplementary Figure S1. Experimental design and delivery of water, honey, and hosts. (a) Flat tubes containing sources of water and/or honey (or none) were used to study different life-history traits of females of Pachycrepoideus vindemmiae (Hymenoptera: Pteromalidae). (b) Flat tubes providing the same water/honey regimens as previous image plus hosts (pupae of spotted-wing drosophila, Drosophila suzukii). (c) Lateral view of the screen cap used to cover the water source and prevent wasp from drinking water while allowing vaporous water to dissipate within the tube. (d) Superior view of the screen cap.

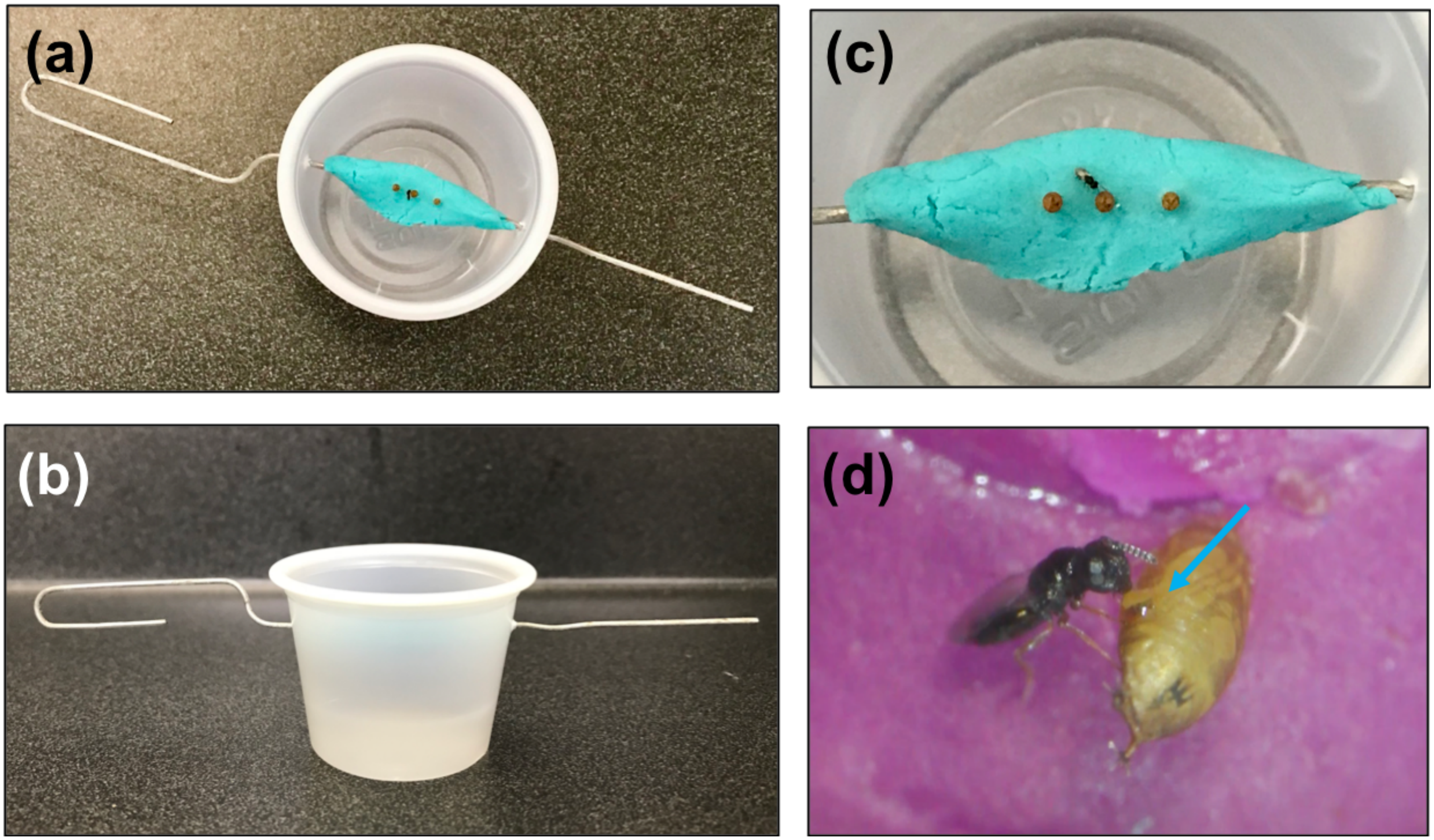

Supplementary Figure S2. Arena used to study the effects of water on the host-feeding behavior of females of Pachycrepoideus vindemmiae (Hymenoptera: Pteromalidae) on pupae of spotted-wing drosophila (SWD), Drosophila suzukii. (a) Superior view. (b) Lateral view. (c) Detail of a P. vindemmiae female approaching a SWD pupa. (d) Host-feeding of P. vindemmiae on the bled-out hemolymph (arrow) of a SWD pupa.

Supplementary Table S1. Results of the normality tests (Shapiro-Wilk, D'Agostino & Pearson, and Kolmogorov-Sminorv's, α=0.05) applied to various data sets collected to determine the effect of water, honey, water + honey, and fasting (no water, no honey) on life-history traits of females of *Pachycrepoideus vindemmiae* (Hymenoptera: Pteromalidae) reared on pupae of spotted-wing drosophila (SWD), *Drosophila suzukii*.

Normality tests

SWD Emergence

	Water + Honey	Honey	Water	Fasting
"Number of values"	10	14	11	13
Minimum	5.48	2.04	2.01	2.03
"25% Percentile"	8.085	3.818	9.86	3.38
Median	12.49	6.02	13.01	5.41
"75% Percentile"	17.67	9.123	18.62	10.42
Maximum	20.95	14.09	25.53	16.89
Mean	12.82	6.611	14.17	7.038
"Std. Deviation"	5.373	3.468	6.56	4.659
"Std. Error of Mean"	1.699	0.9268	1.978	1.292
"Lower 95% CI of mean"	8.972	4.609	9.765	4.223
"Upper 95% CI of mean"	16.66	8.614	18.58	9.854
Sum	128.2	92.56	155.9	91.5
"D'Agostino & Pearson normality test"				
K2	0.6654	1.361	0.07418	2.711
"P value"	0.7170	0.5065	0.9636	0.2578
Passed normality (a=0.05)?	Yes	Yes	Yes	Yes
"P value summary"	ns	ns	ns	ns
"Shapiro-Wilk normality test"				
W	0.9539	0.9566	0.9777	0.8933
"P value"	0.7145	0.6673	0.9521	0.1082
Passed normality (a=0.05)?	Yes	Yes	Yes	Yes
"P value summary"	ns	ns	ns	ns
"KS normality test"				
"KS distance"	0.1094	0.1369	0.1426	0.1751
"P value"	>0.1000	>0.1000	>0.1000	>0.1000
Passed normality (a=0.05)?	Yes	Yes	Yes	Yes
"P value summary"	ns	ns	ns	ns

Parasitoid Emergence

	Water + Honey	Honey	Water	Fasting
"Number of values"	10	14	11	13
Minimum	59.76	67.6	60.34	67.59
"25% Percentile"	67.24	71.63	63.86	72
Median	72.09	76.03	68.8	77.03
"75% Percentile"	78.61	80.28	75.57	81.38
Maximum	83.71	85.63	80.68	84.44

Mean	72.26	76.14	70.6	76.72
"Std. Deviation"	7.314	5.217	6.775	5.366
"Std. Error of Mean"	2.313	1.394	2.043	1.488
"Lower 95% CI of mean"	67.02	73.12	66.05	73.48
"Upper 95% CI of mean"	77.49	79.15	75.16	79.96
Sum	722.6	1066	776.7	997.4
"D'Agostino & Pearson normality test"				
K2	0.02286	0.3564	0.8655	0.798
"P value"	0.9886	0.8368	0.6487	0.6710
Passed normality (a=0.05)?	Yes	Yes	Yes	Yes
"P value summary"	ns	ns	ns	ns
"Shapiro-Wilk normality test"				
W	0.973	0.9814	0.9533	0.9632
"P value"	0.9168	0.9819	0.6858	0.8016
Passed normality (a=0.05)?	Yes	Yes	Yes	Yes
"P value summary"	ns	ns	ns	ns
"KS normality test"				
"KS distance"	0.1343	0.112	0.1505	0.1155
"P value"	>0.1000	>0.1000	>0.1000	>0.1000
Passed normality (a=0.05)?	Yes	Yes	Yes	Yes
"P value summary"	ns	ns	ns	ns

Miscellaneous attacks

	Water + Honey	Honey	Water	Fasting
"Number of values"	10	14	11	13
Minimum	3.4	2.8	2.1	4.9
"25% Percentile"	5.35	7.45	5.9	6.8
Median	6.9	12.9	7	11.1
"75% Percentile"	9.55	16.15	15.4	16.2
Maximum	11.2	21.5	19.5	22.9
Mean	7.211	12.19	9.627	12.03
"Std. Deviation"	2.523	5.68	5.615	5.567
"Std. Error of Mean"	0.8409	1.518	1.693	1.544
"Lower 95% CI of mean"	5.272	8.906	5.855	8.667
"Upper 95% CI of mean"	9.15	15.47	13.4	15.39
Sum	64.9	170.6	105.9	156.4
"D'Agostino & Pearson normality test"				
K2	0.227	0.5214	1.439	0.8013
"P value"	0.8927	0.7705	0.4870	0.6699
Passed normality (a=0.05)?	Yes	Yes	Yes	Yes
"P value summary"	ns	ns	ns	ns
"Shapiro-Wilk normality test"				

W	0.9767	0.9653	0.914	0.9539
"P value"	0.9450	0.8083	0.2715	0.6583
Passed normality (a=0.05)?	Yes	Yes	Yes	Yes
"P value summary"	ns	ns	ns	ns
"KS normality test"				
"KS distance"	0.1368	0.1368	0.2255	0.134
"P value"	>0.1000	>0.1000	>0.1000	>0.1000
Passed normality (a=0.05)?	Yes	Yes	Yes	Yes
"P value summary"	ns	ns	ns	ns

Parasitoid Offspring Mortality

	Water + Honey	Honey	Water	Fasting
"Number of values"	10	14	11	13
Minimum	0	0	0	0
"25% Percentile"	0.56	0.5575	0.56	0
Median	1.15	1.69	1.14	0.57
"75% Percentile"	2.878	3.35	1.7	1.725
Maximum	3.91	5.03	3.69	3.41
Mean	1.494	1.93	1.176	1.045
"Std. Deviation"	1.299	1.529	1.048	1.122
"Std. Error of Mean"	0.4107	0.4087	0.3161	0.3111
"Lower 95% CI of mean"	0.565	1.047	0.4721	0.3677
"Upper 95% CI of mean"	2.423	2.813	1.881	1.723
Sum	14.94	27.02	12.94	13.59
"D'Agostino & Pearson normality test"				
K2	1.855	1.053	6.902	3.075
"P value"	0.3955	0.5907	0.0317	0.2149
Passed normality (a=0.05)?	Yes	Yes	No	Yes
"P value summary"	ns	ns	*	ns
"Shapiro-Wilk normality test"				
W	0.8629	0.9451	0.8765	0.8532
"P value"	0.0825	0.4870	0.0938	0.0314
Passed normality (a=0.05)?	Yes	Yes	Yes	No
"P value summary"	ns	ns	ns	*
"KS normality test"				
"KS distance"	0.3015	0.1312	0.1979	0.2144
"P value"	0.0104	>0.1000	>0.1000	>0.1000
Passed normality (a=0.05)?	No	Yes	Yes	Yes
"P value summary"	*	ns	ns	ns

Natural SWD Death

	Water + Honey	Honey	Water	Fasting
"Number of values"	10	14	11	13
Minimum	6	5.84	6.02	5.9
"25% Percentile"	6.07	5.96	6.04	6.02
Median	6.12	6.04	6.16	6.08

"75% Percentile"	6.26	6.22	6.32	6.23
Maximum	6.47	6.64	6.38	6.25
Mean	6.166	6.106	6.17	6.106
"Std. Deviation"	0.1424	0.2064	0.1306	0.1169
"Std. Error of Mean"	0.04502	0.05516	0.03938	0.03241
"Lower 95% CI of mean"	6.064	5.987	6.082	6.036
"Upper 95% CI of mean"	6.268	6.225	6.258	6.177
Sum	61.66	85.48	67.87	79.38
"D'Agostino & Pearson normality test"				
K2	3.219	8.093	1.684	0.8118
"P value"	0.2000	0.0175	0.4309	0.6664
Passed normality (a=0.05)?	Yes	No	Yes	Yes
"P value summary"	ns	*	ns	ns
"Shapiro-Wilk normality test"				
W	0.9102	0.896	0.8856	0.9139
"P value"	0.2823	0.0986	0.1226	0.2073
Passed normality (a=0.05)?	Yes	Yes	Yes	Yes
"P value summary"	ns	ns	ns	ns
"KS normality test"				
"KS distance"	0.2271	0.1963	0.2038	0.1807
"P value"	>0.1000	>0.1000	>0.1000	>0.1000
Passed normality (a=0.05)?	Yes	Yes	Yes	Yes
"P value summary"	ns	ns	ns	ns

Supplementary Table S2. Results of the homoscedasticity tests (Barllet and Brown-Forsythe's, α=0.05) applied to various data sets collected to determine the effect of water, honey, water + honey, and fasting (no water, no honey) on life-history traits of females of *Pachycrepoideus vindemmiae* (Hymenoptera: Pteromalidae) reared on pupae of spotted-wing drosophila (SWD), *Drosophila suzukii*.

Homoscedasticity tests

	SWD Emergence			
"Data sets analyzed"	Water+Honey	Honey	Water	Fasting
	"Brown-Forsythe test"			
" F (DFn, DFd)"	"1.397 (3, 44)"			
" P value"	0.2564			
" P value summary"	ns			
" Are SDs signif. different (P < 0.05)?"	No			
	"Bartlett's test"			
" Bartlett's statistic (corrected)"	4.583			
" P value"	0.2050			
" P value summary"	ns			
" Are SDs signif. different (P < 0.05)?"	No			
	Parasitoid Emergence			
"Data sets analyzed"	Water+Honey	Honey	Water	Fasting
"Brown-Forsythe test"				
" F (DFn, DFd)"	"0.6163 (3, 44)"			
" P value"	0.6081			
" P value summary"	ns			
" Are SDs signif. different (P < 0.05)?"	No			
"Bartlett's test"				
" Bartlett's statistic (corrected)"	1.778			
" P value"	0.6198			
" P value summary"	ns			
" Are SDs signif. different (P < 0.05)?"	No			
	Miscellaneous attacks			
"Data sets analyzed"	Water+Honey	Honey	Water	Fasting
	"Brown-Forsythe test"			
" F (DFn, DFd)"	"1.471 (3, 44)"			
" P value"	0.2357			
" P value summary"	ns			
" Are SDs signif. different (P < 0.05)?"	No			
	"Bartlett's test"			
" Bartlett's statistic (corrected)"	5.684			
" P value"	0.1280			
" P value summary"	ns			
" Are SDs signif. different (P < 0.05)?"	No			
	Parasitoid Offspring Mortality			
"Data sets analyzed"	Water+Honey	Honey	Water	Fasting

	"Brown-Forsythe test"			
" F (DFn, DFd)"	"0.6783 (3, 44)"			
" P value"	0.5700			
" P value summary"	ns			
" Are SDs signif. different (P < 0.05)?"	No			
	"Bartlett's test"			
" Bartlett's statistic (corrected)"	1.945			
" P value"	0.5840			
" P value summary"	ns			
" Are SDs signif. different (P < 0.05)?"	No			
	Natural SWD Death			
"Data sets analyzed"	Water+Honey	Honey	Water	Fasting
	"Brown-Forsythe test"			
" F (DFn, DFd)"	"0.6456 (3, 44)"			
" P value"	0.5899			
" P value summary"	ns			
" Are SDs signif. different (P < 0.05)?"	No			
	"Bartlett's test"			
" Bartlett's statistic (corrected)"	4.666			
" P value"	0.1980			
" P value summary"	ns			
" Are SDs signif. different (P < 0.05)?"	No			

Supplementary Video S1. Female of *Pachycrepoideus vindemmiae* (Hymenoptera: Pteromalidae) withdrawing her ovipositor from a pupae of spotted-wing drosophila (SWD), *Drosophila suzukii*, and feeding on a drop of hemolymph that extrudes from it.
